# The Effects of LED Light Spectra and Intensities on Plant Growth

**DOI:** 10.3390/plants11151911

**Published:** 2022-07-23

**Authors:** Valeria Cavallaro, Rosario Muleo

**Affiliations:** 1 Institute of BioEconomy (IBE), National Research Council of Italy, 95126 Catania, Italy; 2 Tree Physiology and Fruit Crop Biotechnology Laboratory, Department of Agriculture and Forest Sciences (DAFNE), University of Tuscia, 01100 Viterbo, Italy

Light is an electromagnetic radiation that occurs in a narrow range of over an extremely wide range of wavelengths, from gamma rays with wavelengths to radio waves measured in meters. Light detected by human eyes and plant photoreceptors is a group of wavelengths from about 700 nanometers (nm) for red light down to about 400 nm for violet light: the visible light. However, even the wavelengths form the spectral regions adjacent to the visible ones are referred to as light also, infrared at the red-light and ultraviolet at the violet light end. 

We know that light is the source of energy for the primary sustenance process of life on our earth planet: the photosynthesis which is life’s adopted strategy for capturing and incorporating energy, and under this context in which light is primarily experienced, explored and exploited. However, anyone knows that light is necessary to the aesthetic appreciation of the visual world, and through the sense of sight, light is a primary tool for perceiving the world and communicating within it. Plants also perceive information from the ambient and communicate with other organism using the light and have developed a plethora of photoreceptors that permit the communication with the surrounding ambient.

The physical properties of light as spectral quality, irradiance, intensity, and photoperiod play a deep role on the morphogenesis, growth, and metabolism of many biochemical pathways in plants.

Nowadays, Light-Emitting Diodes (LEDs) have been demonstrated to offer interesting prospects for use in plant lighting designs in controlled environment agriculture (greenhouses) and growth chambers for in vitro cultures. As compared to the previously used light sources, LEDs possess advantages such as wavelength specificity, less heat radiation, longest durability, much lower power consumption, and the possibility to manipulate the spectral qualities of the emitted light.

In high-technology greenhouses (for instance vertical agriculture) artificial light may assume both assimilative and control function. In the first case, light provides the opportunity to optimize photosynthetic efficiency under low (high latitudes) or short day (winter months) solar radiation, enhancing or accelerating the efficiency of plant production. The control activity of light, on the other hand, has the function of guiding growth and development e.g., by promoting changes in the morphology of the plant (e.g., elongation of the stem, branching), or the internal rhythms as the transition from one stage of development to the next (e.g., from vegetative to reproductive), or the synthesis and accumulation of plant metabolites to adapt the plant to adverse environmental conditions, increasing plant fitness, and the nutraceutical properties of the products.

In vitro culture is regulated by different factors, and among them light is the most important. LED illumination system for in vitro cultures should provide light in the spectral region that is involved in photosynthesis and photomorphogenic responses without wasting energy on non-productive wavelengths. The combined effects of light and growth regulators or other components of the culture media is another important issue. Even on in vitro cultures, LED light may regulate gene expression and physiological behaviour that in turn influences metabolite production. 

In this special issue, many of the light concerns had been addressed. Very briefly, the effects on biomass production and photosynthetic efficiency of different light spectra (induced by LEDs) have been reported on *Lactuca sativa* L. [1], *Cucumis sativus* seedlings [2], *Ocimum basilicum* L. [3], two microgreens (*Amaranthus tricolor* L. and *Brassica rapa* L. subsp. oleifera (DC.) Metzg) [4], *Glycine max* (L.) Merr.) [5] and *Medicago Sativa* L. seedlings [6]. The influence of light intensity on *Lactuca sativa* and *Brassica rapa* var. *nipposinica* in vertical farms [7] and of daytime or edge-of-daytime intra-canopy illumination on the fruit set of *Capsicum annuum* [8] were also studied. 

Two papers covered the topic of new technologies (LED-Sourced CoeLux^®^System) [9] and of a new (IoT-Enabled) systems to control light [10].

Interestingly, one article concerned the interactions between light quality and plant pathogens [11]. Two articles regarded some important physiological traits regulation by light, and in particular the effects of supplemental lighting spectra given to widen photoperiod on *Solanum lycopersicum* L. [12] and/or night interrupting on *Chrysanthemum* [13].

The effects of lighting on the production of secondary metabolites in confined environment and in vitro is attracting a growing attention by the researchers. In this special issue, seven articles deal with the influence of LED lighting on important aromatic components in *Mentha canadensis* L. [14], in *Thymus vulgaris* L. [15], and on some nutraceutical and pharmacological components of *Scutellaria baicalensis* [16], *Triticum aestivum* L., *Hordeum vulgare* sprouts [17], *Fritillaria cirrhosa* D. Don, [18], different in vitro cultures [19] and two microgreens [4].

One paper concerned the effects of adding Silicon-Containing Fertilizer [20] on light stress. Finally, a complete review concerning Light and Plant Growth Regulators on in vitro proliferation have been presented [21].

Even if lighting conditions represent an important tool to enhance plant productivity in confined environment, and many issues need still to be explored in this view, this special issue represents an overview of some of the most internationally studied topics.

## References

[1] Tarakanov I.G., Tovstyko D.A., Lomakin M.P., Shmakov A.S., Sleptsov N.N., Shmarev A.N., Litvinskiy V.A., Ivlev A.A. (2022). Effects of Light Spectral Quality on Photosynthetic Activity, Biomass Production, and Carbon Isotope Fractionation in Lettuce, *Lactuca sativa* L. Plants. Plants.

[2] Claypool N.B., Lieth J.H. (2021). Green Light Improves Photosystem Stoichiometry in Cucumber Seedlings (Cucumis sativus) Compared to Monochromatic Red Light. Plants.

[3] Rahman M.M., Vasiliev M., Alameh K. (2021). LED Illumination Spectrum Manipulation for Increasing the Yield of Sweet Basil (*Ocimum basilicum* L.). Plants.

[4] Toscano S., Cavallaro V., Ferrante A., Romano D., Patané C. (2021). Effects of different light spectra on final biomass production and nutritional quality of two microgreens. Plants.

[5] Hitz T., Graeff-Hönninger S., Munz S. (2020). Modelling of Soybean (*Glycine max* L. Merr.) Response to Blue Light Intensity in Controlled Environments. Plants.

[6] Tang W., Guo H., Baskin C.C., Xiong W., Yang C., Li Z., Song H., Wang T., Yin J., Wu X. (2022). Effect of Light Intensity on Morphology, Photosynthesis and Carbon Metabolism of Alfalfa (Medicago Sativa) Seedlings. Plants.

[7] Jayalath T.C., van Iersel M.W. (2021). Canopy Size and Light Use Efficiency Explain Growth Differences between Lettuce and Mizuna in Vertical Farms. Plants.

[8] Tiwari V., Kamara I., Ratner K., Many Y., Lukyanov V., Ziv C., Gilad Z., Esquira I., Charuvi D. (2022). Daytime or Edge-of-Daytime Intra-Canopy Illumination Improves the Fruit Set of Bell Pepper at Passive Conditions in the Winter. Plants.

[9] Beatrice P., Terzaghi M., Chiatante D., Scippa G.S., Montagnoli A. (2021). Morpho-Physiological Responses of *Arabidopsis thaliana* L. to the LED-Sourced CoeLux^®^System. Plants.

[10] Afzali S., Mosharafian S., van Iersel M.W., Mohammadpour Velni J. (2021). Development and Implementation of an IoT-Enabled Optimal and Predictive Lighting Control Strategy in Greenhouses. Plants.

[11] Sgamma T., Forgione I., Luziatelli F., Iacona C., Mancinelli R., Thomas B., Ruzzi M., Muleo R. (2021). Monochromic radiations provided by light emitted diode (LED) modulate infection and defense response to fire blight in pear trees. Plants.

[12] Lanoue J., Thibodeau A., Little C., Zheng J., Grodzinski B., Hao X. (2021). Light Spectra and Root Stocks Affect Response of Greenhouse Tomatoes to Long Photoperiod of Supplemental Lighting. Plants.

[13] Park Y.G., Jeong B.R. (2020). How Supplementary or Night-Interrupting Low-Intensity Blue Light Affects the Flower Induction in Chrysanthemum, A Qualitative Short-Day Plant. Plants.

[14] Ueda T., Murata M., Yokawa K. (2021). Single Wavelengths of LED Light Supplement Promote the Biosynthesis of Major Cyclic Monoterpenes in Japanese Mint. Plants.

[15] Tabbert J.M., Schulz H., Krähmer A. (2021). Increased Plant Quality, Greenhouse Productivity and Energy Efficiency with Broad-Spectrum LED Systems: A Case Study for Thyme (*Thymus vulgaris* L.). Plants.

[16] Yeo H.-J., Park C.-H., Park S.-Y., Chung S.-O., Kim J.-K., Park S.-U. (2021). Metabolic Analysis of Root, Stem, and Leaf of Scutellaria baicalensis Plantlets Treated with Different LED Lights. Plants.

[17] Muthusamy M., Kim J.H., Kim S.H., Kim J.Y., Heo J.W., Lee H., Lee K.-S., Seo W.D., Park S., Kim J.A. (2020). Changes in Beneficial C-glycosylflavones and Policosanol Content in Wheat and Barley Sprouts Subjected to Differential LED Light Conditions. Plants.

[18] Chen C.-C., Lee M.-R., Wu C.-R., Ke H.-J., Xie H.-M., Tsay H.-S., Agrawal D.C., Chang H.-C. (2020). LED Lights Affecting Morphogenesis and Isosteroidal Alkaloid Contents in Fritillaria cirrhosa D. Don—An Important Chinese Medicinal Herb. Plants.

[19] Hashim M., Ahmad B., Drouet S., Hano C., Abbasi B.H., Anjum S. (2021). Comparative Effects of Different Light Sources on the Production of Key Secondary Metabolites in Plants In Vitro Cultures. Plants.

[20] Semenova N.A., Smirnov A.A., Grishin A.A., Pishchalnikov R.Y., Chesalin D.D., Gudkov S.V., Chilingaryan N.O., Skorokhodova A.N., Dorokhov A.S., Izmailov A.Y. (2021). The Effect of Plant Growth Compensation by Adding Silicon-Containing Fertilizer under Light Stress Conditions. Plants.

[21] Cavallaro V., Pellegrino A., Muleo R., Forgione I. (2022). Light and Plant Growth Regulators on In Vitro Proliferation. Plants.

